# The Potential of CO_2_ Hydrogenation to Produce e‑Fuels: Thermodynamic and Techno-economic Analysis

**DOI:** 10.1021/acs.energyfuels.6c00395

**Published:** 2026-04-24

**Authors:** Ivan L. Amorim, A. Catarina Faria, Cláudio Rocha

**Affiliations:** † LEPABE, ALiCE, Faculty of Engineering, University of Porto, Rua Dr. Roberto Frias, Porto 4200-465, Portugal; ‡ Fraunhofer Portugal AWAM - Research Center for Advanced Water, Energy and Resource Management, 616347 Régia Douro Park, Parque de Ciência e Tecnologia, Vila Real 5000-033, Portugal; § Centre for the Research and Technology of Agroenvironmental and Biological Sciences, CITAB, Inov4Agro, Universidade de Trás-os-Montes e Alto Douro, UTAD, Quinta de Prados, Vila Real 5000-801, Portugal

## Abstract

Since the 19th century, petroleum has underpinned societal development and industrial progress, serving as a primary source of heating, power, and transportation fuels. However, its detrimental effects on the environment and urban areas have highlighted the urgent need to transition toward more sustainable energy alternatives. CO_2_ hydrogenation to electrofuels (i.e., e-fuels) emerges as a promising pathway for cleaner energy production. This study focuses on the thermodynamic and techno-economic aspects for 12 different e-fuels: methane, ethane, propane, butane, pentane, gasoline, kerosene, diesel, methanol, ethanol, butanol, and dimethyl ether. For thermodynamic assessment, Aspen Plus V14 software was used to simulate an equilibrium reactor fed with pure CO_2_ and H_2_ streams under different temperatures, pressures, and H_2_/CO_2_ ratios. The results showed an increase in fuel fractional yield and CO_2_ conversion with higher pressure and lower temperatures. The initial economic analysis considered the determination of the production phase rentability parameter (i.e., α); a value above 1 indicates that fuel sales surpass the combined costs of green hydrogen and operational expenditure. Excluding capital expenditure and assuming fuel prices equivalent to conventional production methods, the analysis revealed potential rentability for gasoline under both best- (200 °C, 50 bar) and worst-case (350 °C, 20 bar) conditions, corresponding to the highest and lowest yields, respectively, whereas diesel was found to be profitable only under the best-case scenario. When capital expenditure and parameters such as workforce were considered, the initial investment was estimated at approximately 7–8 million euros, excluding additional costs associated with gas purification, transport, and storage. The cumulative cash flow showed progressive decreases, suggesting the unviability of the project under current conditions. However, a sensitivity analysis of hydrogen costs indicated potential feasibility for gasoline and diesel production if hydrogen prices drop to half their current levels.

## Graphical abstract

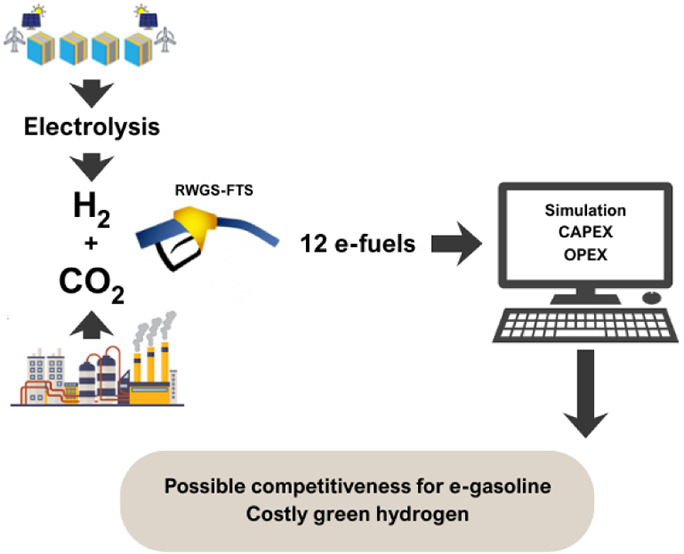

## 1 Introduction

Petroleum, at the turn of the 20th century, was plentiful; in fact, society and business were built around this fuel, which supplied most of the energy needs for these sectors. New generations face the challenge of re-engineering a petroleum-dependent world into one driven by sustainable alternatives, while also contending with the health and lifestyle consequences of fossil fuel reliance. Global demand for oil products has continued to grow: in 2023, global demand reached ∼102.2 million barrels per day, a staggering 16.2 billion liters per day, and the forecast for 2050 is 120 million barrels per day (19.1 billion liters per day).^1^ In this context, the development of renewable fuels offers not only environmental benefits but also the potential for improved energy efficiency.^2^

The Paris Agreement (2015) established a global consensus to address climate change by limiting the rise in global average temperature to below 2 °C above preindustrial levels, aiming to keep this value even lower, at 1.5 °C.^3^ To achieve this, global carbon emissions have to fall by 45% by 2030. However, in 2024, atmospheric CO_2_ concentrations reached an average of 422.8 ppm—an increase of 3.8 ppm compared to 2023 and the largest one-year increase on record.^4^ Moreover, projections for the end of the century indicate concentrations of around 560 ppm.^5^ The Paris Agreement set an ambitious goal, which has prompted countries to accelerate the transition to renewable energy sources. Global tensions between countries like Russia and Ukraine have heightened the urgency of shifting to cleaner energy sources, which have proven to be more reliable and economically viable.^3^ However, renewable resources like wind and solar are inherently intermittent and variable, producing energy only under specific weather conditions and times. This intermittency creates supply volatility, which in turn causes price fluctuations and challenges in meeting demand. Along with renewable energy, other forms of energy generation, such as electrofuels, have emerged to support the transition toward a fossil fuel-independent society and help to balance the intermittency of electricity production.

Electrofuels, or e-fuels, are hydrogen-based synthetic fuels produced using electricity (ideally from renewable sources), water, and carbon dioxide to form hydrocarbon compounds (HC). Conventional fuels like gasoline and diesel can be produced as e-fuels, which means fuel production based on carbon capture could substitute fossil oil- and gas-based fuels. Interest in e-fuels has grown largely due to their compatibility with existing internal combustion engines and fueling infrastructure, not requiring a significant investment in new systems. Aviation and deep-sea shipping, being long-distance transport modes, are limited in terms of electrification opportunities, so the energy-dense e-fuels might be of special interest.^6^ The current landscape of e-fuel research is largely centered on catalytic development or thermodynamics of the production of single fuels (often focusing on methane,^7^ methanol,^8^ and sustainable aviation fuel—e-kerosene^9^), while limited attention is given to comparison of thermodynamic aspects or economic assessments across a wide range of fuels under consistent assumptions. These existing studies provide limited insight into which fuels might offer the greatest thermodynamic advantage or exhibit superior economic robustness when comparing electrofuel production options with conventional fossil fuel exploitation. Thus, this study aims to conduct a thermodynamic equilibrium evaluation and a techno-economic analysis relying on a universal flowsheet for direct cost comparison purposes.

### 1.1 E-Fuels

The catalytic hydrogenation of CO_2_ into chemical feedstocks has attracted significant research interest as a promising technology for recycling CO_2_ emissions from power plants.^10^ These chemical feedstocks can be carbon-based fuels produced from raw materials such as green H_2_, CO, and CO_2_: electrofuels or e-fuels. Carbon dioxide can be derived from several sources, depending on the industry and segment, as described in Table 1.

**Table 1:** Typical CO_2_ Concentrations in the Gas Streams of Various Sources^a^

Industry field	Segment	CO_2_ concentration/vol %
Biomass	Biomass fermentation	15–50
	Biogas upgrading	80–100
	Bioethanol production	≈100
Power generation	Natural gas combustion	3–5
	Petroleum combustion	3–8
Industrial	Cement production	14–33
	Iron and steel production	20–30
	Hydrogen production	15–20
	Methanol production	10
N/A^b^	Direct Air Capture (DAC)	0.04

a Adapted with permission from ref. 11. Copyright 2026 Elsevier.

b N/A: Not applicable.

In the biomass sector, the source composition will depend on the feedstock type and digestion conditions. Power generation plants produce large amounts of CO_2_, but as renewable energy usage rises, it might be of added relevance to shift attention toward industrial point sources or DAC. Cement and iron plants emit high concentrations of this greenhouse gas, making them particularly attractive sources for utilization. In contrast, DAC aims not to mitigate the rise in atmospheric CO_2_ concentration but to actively reduce it. Although DAC technologies have started to be studied, their application remains challenged due to the high costs associated with the very low CO_2_ partial pressure in air.^12^

While H_2_ can be used directly as a fuel, the hydrogenation of carbon dioxide enables the production of a broader range of fuels, including direct substitutes for conventional fossil fuels. Methane, ethanol, methanol, and various hydrocarbons such as paraffins, olefins, and dimethyl ether (DME) are some examples of e-fuels that can be formed through catalytic hydrogenation of CO_2_.

### 1.2 Catalytic Hydrogenation of CO_2_

The chemical reactions to produce paraffins, olefins, alcohols, and dimethyl ether (DME) are described in Table 2. In these equations, *n* is the number of carbon atoms, which come from each CO_2_ molecule that contributes to the reaction in the carbonated product. The notable reverse water–gas shift (RWGS) and the Fischer–Tropsch synthesis (FTS) reactions constitute the most usual indirect CO_2_ hydrogenation route, in which the raw material is converted into CO, which then is converted into hydrocarbons (such as paraffins or olefins).^13^ In turn, other studies consider the direct hydrogenation of CO_2_, as seen in Table 2 (e.g., the Sabatier reaction for methanation—Eq 4). Table 2 also provides the enthalpies of reaction, indicating whether each process is exothermic or endothermic. For exothermic processes, an increase in *n* and, consequently, in the produced fuel’s carbon chain length leads to progressively more intense exothermicity.

**Table 2:** Main Reactions Involved in CO_2_ Hydrogenation

Equation number	Reaction equation	Δ*H*_r,298 K_/kJ·mol^–1^	Formation of
1	1 $CO_{2}+H_{2}⇌CO+H_{2}O$	41	CO (RWGS reaction)
2	2 $CO+1/2O_{2}⇌CO_{2}$	–283	CO_2_ (CO oxidation)
3	3 $CO_{2}+4H_{2}⇌CH_{4}+2H_{2}O$	–165	CH_4_ (Sabatier)
4	4 $nCO_{2}+\left(3n+1\right)H_{2}⇌C_{n}H_{2n+2}+2nH_{2}O$	<0	n-Paraffins (FTS reaction)
5	5 $nCO+\left(2n+1\right)H_{2}⇌C_{n}H_{2n+2}+nH_{2}O$	<0	
6	6 $nCO_{2}+3nH_{2}⇌C_{n}H_{2n}+2nH_{2}O\;\left(n\geq 2\right)$	<0	n-Olefins (FTS reaction)
7	7 $nCO+2nH_{2}⇌C_{n}H_{2n}+nH_{2}O\;\left(n\geq 2\right)$	<0	
8	8 $CO_{2}+3H_{2}⇌CH_{3}OH+H_{2}O$	–49	Methanol
9	9 $nCH_{3}OH⇌C_{n}H_{2n}+nH_{2}O\;\left(n\geq 2\right)$	<0	n-Olefins (Methanol route)
10	10 $nCO_{2}+3nH_{2}⇌C_{n}H_{2n+1}OH+\left(2n-1\right)H_{2}O$	<0	n-Alcohols
11	11 $nCO+2nH_{2}⇌C_{n}H_{2n+1}OH+\left(n-1\right)H_{2}O$	<0	
12	12 $2CH_{3}OH⇌CH_{3}OCH_{3}+H_{2}O$	–24	DME (MeOH dehydration & FTS)
13	13 $2CO_{2}+6H_{2}⇌CH_{3}OCH_{3}+3H_{2}O$	–123	

In relation to the catalysts considered for these processes, Kusama et al. have observed that when the reaction is carried out over catalysts such as Rh/SiO_2_ with no precursors or with the precursors CaCl_2_ or MgCl_2_·6H_2_O, methane can be obtained with a selectivity of up to 100%.^10^ At the same time, rhodium-based catalysts modified with additives such as lithium and iron, correspondingly, Rh-Li(1:1)/SiO_2_ ^10^ and Rh-Fe(1:2)/SiO_2_,^14^ can achieve methane selectivities of 63.5% and 34.7%, respectively, while producing ethanol at selectivities of 15.5% and 16%. In addition, Wang et al. also reported ethanol selectivities of 98.2% and 99.8% using CoAlO*_x_-*600 catalysts in tank-type reactors.^15^

Paraffins and olefins of long carbon chains can also be obtained via catalysts with alkali-metal supports, specifically sodium and cobalt or copper. Na-Fe_2_Zn_1_ ^16^ presents a 68.2% selectivity for heavy hydrocarbons, while Na/Fe_3_O_4_ on a ZSM-5 support^17^ yields a value of 75.5%. Additionally, 64.2% C_8+_ hydrocarbons can be obtained by using CoFe-0.81Na in a one-pot FTS reactor.^18^ Mesoporous ZSM-5 support H-meso-ZSM-5-0.5 M on cobalt catalysts has also been tested in hydrogenation via the methanol route, yielding 70% very heavy hydrocarbons containing 12 or more carbon atoms.^19^ A modified FTS route over CuFeO_2_-24 catalysts has also shown great results for C_5+_ hydrocarbons, with a 64.9% selectivity value.^20^

To produce light hydrocarbons, alkali metals have once again shown an excellent performance as additives in iron-based catalysts. The use of 35Fe-7Zr-1Ce-K^21^ and C-2Fe-Zn/K^22^ catalysts proved to selectively produce 80.4% and 83.4% light hydrocarbons, respectively. Fe-Cu(0.17)/K(1.0)^23^ and Na-Fe-Zn^24^ have yielded selectivities of 76% and 42% for light olefins, respectively, in stainless steel and titanium fixed-bed high-pressure reactors via the RWGSFTS route, as described by Eqs 7 and 8. Through the methanol route, the ZnZrO/SAPO-34 catalyst demonstrated a selectivity of 80% for light olefins.^25^

Methanol has also been very worthy of investigation, despite the typically required high-pressure conditions. Indeed, Cu-ZnO/ZrO_2_ ^26^ and platinum-based catalyst Pt_1_@MIL^27^ have achieved methanol selectivities of 83% and 90%, respectively.

Finally, DME has been produced via methanol synthesis and subsequent dehydration over a CuO-ZnO-Al_2_O_3_-ZrO_2_ (12:6:1:1) catalyst under mild operating conditions, reaching a selectivity of 15.8%.^28^

To date, no studies have specifically investigated the selective hydrogenation of CO_2_ to butanol; however, research on the production of higher alcohols in general has been reported. 1 wt % Pt/Co_3_O_4_ has shown great selectivity to C_2+_ alcohols, at 82.5% in a batch reactor, at 200 °C and 80 bar, with a feed H_2_/CO_2_ ratio of 3.^29^

## 2 Methods

### 2.1 Process Simulation

CO_2_ hydrogenation was simulated using the Aspen Plus V14 software. The Soave–Redlich–Kwong (RKSOAVE method) equation of state has been extensively used in calculating phase and reaction equilibrium, and results have been consistent with experiments. Thus, it was used to calculate reaction equilibrium at high pressure.^30^ Table 3 summarizes the chemical species considered in the process simulation, as well as their chemical formulas and unique identification Chemical Abstracts Service (CAS) numbers.^31^

**Table 3:** List of Chemical Species Considered for Process Simulation^31^

Component	Chemical Formula	CAS Number
Water	H_2_O	7732-18-5
Hydrogen	H_2_	1333-74-0
Nitrogen	N_2_	7727-37-9
Carbon monoxide	CO	630-08-0
Carbon dioxide	CO_2_	124-38-9
Methane	CH_4_	74-82-8
Paraffins	C_*n*_H_2*n*+2_	(Various)
Olefins	C_*n*_H_2*n*_ (*n* ≥ 2)	(Various)
Dimethyl ether	CH_3_OCH_3_	115-10-6
Methanol	CH_3_OH	67-56-1
Ethanol	C_2_H_5_OH	64-17-5
Butanol	C_4_H_9_OH	71-36-3

Simulations were performed under pressures, temperatures, and H_2_/CO_2_ ratio ranges currently used in e-fuel research and industry, as summarized in Table 4.

**Table 4:** Conditions Considered in the Process Simulation of Each e-Fuel Synthesis

Main product	*P*/bar	*T*/°C	H_2_/CO_2_	Reference
Methane	250–400	1–30	4	^7^
C_2_–C_5_ paraffins	200–350	10–50	3	^13–22^
Gasoline	200–350	20–50	3	
Kerosene				
Diesel				
Methanol	200–350	50–100	3–4	^8^
Ethanol	250–300	20–50	3–4	^32^
Butanol	250–350	30–100	3–4	^33^
DME	200–300	30–80	3	^34^

The “Sensitivity Analysis” tool was employed to evaluate and optimize the operation conditions, eliminating the need to run thousands of individual simulations.

#### 2.1.1 Basic Approach

As a first and basic approach, a simple simulation configuration was used to calculate the FFE and CO_2_ conversion for the production of several different e-fuels: CO_2_ and H_2_ (“FEED” stream), considering several H_2_/CO_2_ ratios were fed to an RGibbs (“REACTOR”) block at different temperatures and pressures (those referred to in Table 4), which yielded the e-fuels in the “PRODUCTS” stream, as represented in Figure 1.

**Figure 1:**
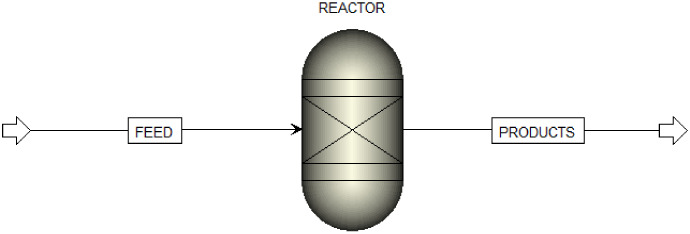
Configuration for chemical equilibrium simulation in Aspen Plus V14 software.

RGibbs, aside from working as an equilibrium reactor, assumes the operation of an isothermal reactor at a user-defined temperature. This reactor model works by simultaneously minimizing the Gibbs free energy and does not require the specification of the reactions and their stoichiometry.^35^ The possible products were introduced to the software: H_2_, CO_2_, H_2_O and the desired e-fuels methane, ethane, propane, butane, pentane, gasoline (in the form of hexane), kerosene (octane), diesel (nonane), methanol, ethanol, butanol, and DME (in the case of methane production, CO formation was also considered since it can be an intermediate in these types of processes). Thus, in each simulation, only one of the e-fuels’ production was considered. Since gasoline, kerosene, and diesel are mixtures of diverse molecules, the simulation was carried out considering the smallest, therefore more thermodynamically stable, molecule of each one. Otherwise, the solution would always converge to the more stable molecule.

RGibbs models’ fragility lies in the difficulty to scientifically validate a near-perfect equilibrium result; CO_2_ hydrogenation to targeted products is a kinetically controlled process influenced by thermodynamics and catalyst selectivity, and RGibbs might neglect the true behavior of catalysts and conversion rate. Mathematically, product distribution of hydrocarbons formed through FTS follows the Anderson–Schulz–Flory distribution, characterized by chain-growth probability.^36^ Other models like RYield and RStoic were not preferred: the stoichiometry of the possible reactions involved is known, thus RYield calculations were not necessary; besides that, there was no need to define reaction extent and conversion since it was expected the determination of the equilibrium compositions in the simulations, justifying the choice of not implementing the RStoic or RYield models.

#### 2.1.2 Integrated Approach

The integrated approach in this work included pressure changers, temperature controllers, and condensers along with an RGibbs reactor and was later used to assess energy requirements and costs associated with each unit operation. Simulations considered a mixture with a composition identical to that of flue gas (80 vol % N_2_ and 20 vol % CO_2_) and pure hydrogen. Even though the typical flue gas composition from natural gas-fired power plants is around 10 vol % CO_2_, 18 vol % H_2_O and 72 vol % N_2_,^37^ it was assumed that the stream was previously treated and water was removed. Flue gas is fed at a constant flow rate of 250 kmol·h^–1^ (i.e., 50 kmol·h^–1^ of CO_2_ and 200 kmol·h^–1^ of N_2_) and pure hydrogen at 150 kmol·h^–1^ (for H_2_/CO_2_ = 3) or 200 kmol·h^–1^ (for H_2_/CO_2_ = 4). Nitrogen was excluded in the basic approach since, as an inert component and stream diluent, it has no impact on yield, normalized by CO_2_ input only, or conversion. However, it does influence the energy requirements associated with pressure and temperature changes. The integrated approach was applied to the pressure and temperature conditions listed Table 4, which yielded the highest and lowest yields. Although the lower yield conditions are less favorable thermodynamically to e-fuel, it is of interest to study these cases from an economic and more realistic operational scenario.

Figure 2 depicts the process flowsheet of the integrated approach: the flue gas (stream “S1”) enters the system at 150 °C and 1 bar,^37^ and it can be compressed through a series of compressor-cooler units. It is important not to exceed a compressor outlet temperature of 200 °C to prevent excessive wear and prolong compressor lifespan;^38^ therefore, intermediate cooling using water at 20 °C is necessary between compression stages. Since each e-fuel is produced at different pressures, not all three compressors depicted in the process flowsheet are necessarily required. Specifically:

- At 1 bar, no compressor or cooler is required.
- At 10–20 bar, 2 compressors and 1 cooler are required.
- At 20–100 bar, all 3 compressors and 2 coolers are required.

**Figure 2:**
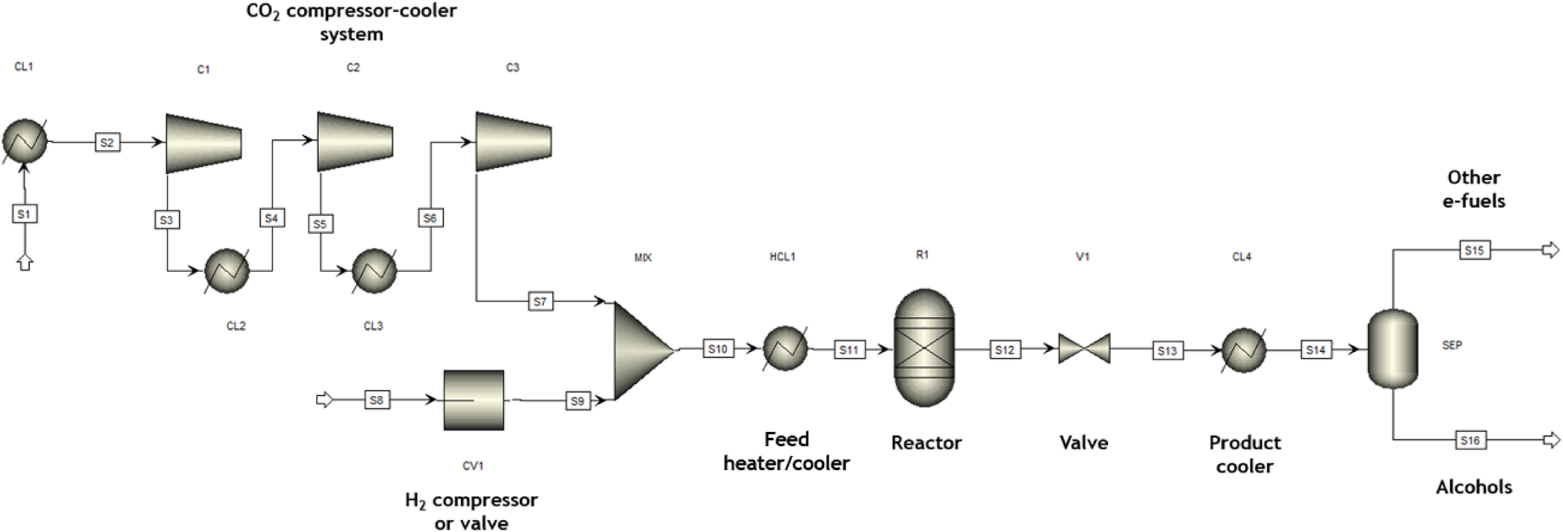
Configuration for the integrated approach in Aspen Plus V14 software.

Hydrogen, represented by stream “S8”, is assumed to be supplied from the outlet of an alkaline electrolyzer. This device normally works at temperatures and pressures ranging from, respectively, 50–80 °C and 15–40 bar.^39^ For this simulation, the worst-case scenario was assumed, i.e., the hydrogen stream enters at 80 °C and 40 bar. Then, “S8” stream is either compressed or expanded depending on the required reaction pressure using the device represented by block “CV1”, which functions either as a compressor or an expansion valve. The resulting stream, “S9”, has both reactants adjusted to the fixed pressure at which the reactor will operate. These streams are then mixed in block “MIX” and the resulting stream, “S10”, is fed to a heat exchanger, “HCL1”, which works either as a heater or a cooler (the latter in the particular case of methanol production—at 200 °C and 100 bar), with the goal of achieving the reaction temperature before being fed to the equilibrium reaction block “R1”. To simplify the simulation and operation, the condensation part of the process happens at atmospheric pressure; the associated pressure drop was promoted using a valve, “V1”. Condensation must occur at a temperature and pressure at which vapor and liquid easily separate. It has been assumed that the condenser operates at atmospheric pressure and varied temperatures, all lower than the reactor temperature. Aspen’s “Sensitivity Analysis” tool was utilized to find the lowest temperature to operate block “CL4” and feed no liquid fraction to the condenser, and to find a low enough temperature at which the condenser can operate to obtain the maximum product possible. Phase separation of the gaseous outlet from the reactor and the last cooler was modeled using an equilibrium flash block as a condenser, capturing the first separation step in CO_2_ hydrogenation products. This block allowed for generalized calculation. A RadFrac column, though more accurate, requires parameters specific to each fuel, restricting the desired comparability of the universal flowsheet.

Hydrogen recycle increases the utilization of green hydrogen, an expensive feedstock, and is commonly implemented in industrial processes, thus being assumed standard in simulation studies. Though providing improved realism, this recycling loop was intentionally excluded, as comparability might have become compromised. This compromise would come from differences in optimal recycle ratios or an inefficiency shift from underusing hydrogen to the need for additional compression and cooling duties. Besides, the equilibrium composition would not change.

#### 2.1.3 Techno-economic Analysis

The first approach to assess the techno-economic viability of e-fuel production considers operating expenditures (OPEX), feedstock costs, and fuel revenues. For this, the energy requirements of the heat exchangers, compressors, and condenser were considered, namely, their net duties (*Q*). An annual operating time of 8000 h yearly, i.e., ca. 333 days, was assumed for each plant. Besides being a commonly used benchmark, it allows for just over 30 days dedicated to maintenance, inspection, sporadic shutdowns, and catalyst renovation or to account for operational variability during the working hours.

The Chemical Engineering Plant Cost Index (CEPCI, *C*_Index_) is applied to update the costs of equipment (*C*_Curr_) to the values of June 2024 (latest publicly available CEPCI – 798.8) in relation to those of a determined year (*C*_Ref_), according to Eq 14.

14 $$C_{\mathrm{Curr}}=C_{\mathrm{Ref}}\cdot \frac{C_{{\mathrm{Index}}_{\mathrm{Curr}}}}{C_{{\mathrm{Index}}_{\mathrm{Ref}}}}$$

The energy cost associated with the compressor’s operation, *C*_EC_, can be estimated by Eq 15 considering an electricity price of 0.0987 €·kWh^–1^, for bihourly operation, reported by EDP.^40^ The Portuguese company’s energy production in 2024 was 95% renewable,^41^ fitting e-fuel production requirements.

15 $$C_{\mathrm{EC}}\left(€\cdot y^{-1}\right)=Q\left(\mathrm{kW}\right)\cdot 8000\left(h\cdot y^{-1}\right)\cdot 0.0987\left(€\cdot {\mathrm{kWh}}^{-1}\right)$$

For heaters, electricity was employed as well, at the same price point. For coolers (Eq 16), the cost of cooling water was set at 0.016 €·m^–3^, which includes the makeup and treatment, as reported in a previous work.^42^ It was assumed that the cooling water entered the heat exchanger at 20 °C (i.e., *T*_CW,in_) and exited at 50 °C (i.e., *T*_CW,out_).

16 CCW(€·y−1)=Q(kW)·0.016(€·m−3)·3600(s·h−1)·8000(h·y−1)ρCW(kg·m−3)·Cp,CW(kJ·kg−1·C−1°)·(TCW,out−TCW,in)(C°)

where ρ_CW_ is the density of cooling water (1000 kg·m^–3^) and *C*_p,CW_ is the specific heat capacity of cooling water (4.18 kJ·kg^–1^·°C^–1^).

The reactor’s energy expenses were not considered, as previous techno-economic analysis has shown that the energy required to keep a constant reactor temperature is negligible compared to the overall energy demand of the industrial plant.^42^

In the condenser block, for the separation of hydrocarbons and DME, chilled water at 4 °C was used, at a cost of 0.02 €·kWh^–1^. For alcohol separation, a refrigeration fluid was employed: at 101 °C the cost was 0.06 €·kWh^–1^, while at 68 °C it was 0.04 €·kWh^–1^. The energy cost associated with the separation process, *C*_ES_, is described in Eq 17.

17 $$C_{\mathrm{ES}}\left(€\cdot y^{-1}\right)=Q\left(\mathrm{kW}\right)\cdot 8000\left(h\cdot y^{-1}\right)\cdot C_{R}\left(€\cdot {\mathrm{kWh}}^{-1}\right)\cdot \frac{C_{{\mathrm{Index}}_{\mathrm{Curr}}}}{C_{{\mathrm{Index}}_{2009}}}$$

where *C*_R_ is the utility cost in 2009,^43^ further updated with the current CEPCI.

The hydrogen cost for this project was set at 3 €·kg^–1^, corresponding to the lower limit of the reported European production cost range.^44^

Based on the hydrogen cost, OPEX, and e-fuel selling prices (Table 5), a rentability parameter for the production phase was calculated to evaluate the potential for profit in CO_2_ hydrogenation to certain e-fuels. Eq 18 defines this parameter, α, as the ratio between the annual fuel revenue and the sum of annual hydrogen cost and operational expenditures. Despite efforts to standardize fuel locations for pricing purposes, complete data was not available for all cases.

**Table 5:** Current Conventional Fuel Prices in Various Locations

Fuel	Price/€·kg^–1^	Location
Methane	0.88 ^45^	Portugal
Ethane	0.15 ^46^	United States
Propane	0.55 ^47^	Europe
Butane	0.58 ^48^	Belgium
Pentane	1.09 ^49^	China
Gasoline	2.09 ^50^	Portugal
Kerosene	1.04 ^51^	(World average)
Diesel	1.65 ^50^	Portugal
Methanol	0.63 ^52^	Europe
Ethanol	0.82 ^53^	Europe
Butanol	1.17 ^54^	China
DME	1.70 ^55^	Europe

If α ≤ 1, the process is considered economically unviable when only operating expenditures (OPEX) and feedstock costs are accounted for. In contrast, when α > 1, the process has the potential to be economically feasible, indicating that CAPEX should be considered and a more complete economic assessment performed using the project assessment tool developed by the Agency for Competitiveness and Innovation (IAPMEI).

18 $$\alpha \left[{€}_{\mathrm{Fuel}}\cdot {\left({€}_{H_{2}}+{€}_{\mathrm{OPEX}}\right)}^{-1}\right]=\frac{C_{\mathrm{Fuel}}\left({€}_{\mathrm{Fuel}}\cdot y^{-1}\right)}{C_{H_{2}}\left({€}_{H_{2}}\cdot y^{-1}\right)+C_{\mathrm{Op}}\left({€}_{\mathrm{OPEX}}\cdot y^{-1}\right)}$$

Therefore, for process cases wherein α > 1, the economic analysis was extended to assess the project’s profitability 10 years postinvestment, taking into account CAPEX, e-fuel revenue, OPEX, hydrogen cost, invested capital, production costs, workforce, and utilities.

The cost of a compressor, *C*_C_, was estimated using Eq 19, which is applicable to reciprocating compressors.

19 $$C_{C}\left(€\right)=e^{7.6084+0.80\cdot \ln\left(P_{\mathrm{out}}\right)}$$

where *P*_out_ is the discharge pressure.

The six-tenths rule of thumb allows for direct estimation of the cost of a sized equipment from a reference cost and respective capacity. Eqs 20 and 21 were used to determine, respectively, the cost of the reactor, i.e., *C*_Re_, and the heat exchangers, i.e., *C*_HX_, from reference values *C*_Re,Ref_ and *C*_HX,Ref_, respectively. Both current values for reactor molar flow and exchanger net duty, i.e., *F*_Re_ and *Q*_HX_, and reference capacities for the aforesaid variables, *F*_RE,Ref_ and *Q*_HX,Ref_ are also accounted for.

20 CRe=CRe,Ref·(FReFRe,Ref)0.6

21 $$C_{\mathrm{HX}}=C_{\mathrm{HX},\mathrm{Ref}}\cdot {\left(\frac{Q_{\mathrm{HX}}}{Q_{\mathrm{HX},\mathrm{Ref}}}\right)}^{0.6}$$

For the reactor, the reference values considered in this analysis were taken from a study on one-step conversion of CO_2_ to DME, namely *C*_Re,Ref_ of 239 k€ and *F*_Re,Ref_ of 3526 kg·h^–1^.^56^ In turn, the reference values of the heat exchanger were based on Rocha et al.,^42^ with reference cost and heat duty of, respectively, 28.4 k€ and 500 kW.

To evaluate the projects’ profitability, the net present value (NPV), the internal rate of return (IRR), and the payback time were assessed. The NPV was calculated at the end of a 10-year period and set to zero for IRR calculation. A capital cost of 12% and a corporate income tax of 25% were considered, along with a 10-year amortization period. Moreover, an iterative process was performed to determine the minimum selling price that would yield a positive cash flow after 10 years. Sensitivity analysis was also conducted to evaluate the effect of reducing the hydrogen cost to 1 €·kg^–1^, 1.5 €·kg^–1^, 2 €·kg^–1^, and 2.5 €·kg^–1^ on the cash flow.

#### 2.1.4 Sensitivity Analysis

The sensitivity analysis tool was used to study the effect of temperature, pressure, and H_2_/CO_2_ molar feed ratios on fuel fractional yield (*Y*_*i*_, Eq 22) and CO_2_ conversion ( $X_{{\mathrm{CO}}_{2}}$ , Eq 23). The ranges of temperature, pressure, and H_2_/CO_2_ molar ratios were simulated according to Table 4. Temperature and pressure were varied using step sizes of 10 °C and 2 bar, respectively (except in the methane simulation, where the initial pressure step was from 1 to 2 bar).

22 $$Y_{i}\left({\mathrm{mol}}_{i,\mathrm{out}}\cdot {\mathrm{mol}}_{{\mathrm{CO}}_{2},\mathrm{in}}^{-1}\right)=\frac{F_{i,\mathrm{out}}}{F_{{\mathrm{CO}}_{2},\mathrm{in}}}$$

23 $$X_{{\mathrm{CO}}_{2}}\left(\%\right)=\frac{F_{{\mathrm{CO}}_{2},\mathrm{in}}-F_{{\mathrm{CO}}_{2},\mathrm{out}}}{F_{{\mathrm{CO}}_{2},\mathrm{in}}}\cdot 100$$

where *F*_*i*,out_ and *F*_*i*,in_ are the molar flow of species *i* at the outlet and inlet, respectively.

This tool was also applied to determine the optimal operating conditions that maximize the yields. Finally, for techno-economic analysis, a sensitivity test was performed to assess the effect of hydrogen cost variations, the largest expense, on the overall rentability.

## 3 Results and Discussion

### 3.1 Basic Approach—Thermodynamic Analysis

Figures 3 and 4 show that all CO_2_ valorization routes follow the same trends: CO_2_ conversion and fractional yield increase with decreasing temperature due to the exothermic nature of the reactions listed in Table 2, and with increasing pressure, in accordance with the reduction in the total number of moles from reactants to products. Each plane in both graphs represents a fixed H_2_/CO_2_ value, varying from 1 (inferior plane) to 5 (superior plane), with unit increments, allowing for the analysis of the effect of hydrogen in the conversion of CO_2_ and fractional fuel yield. As a result, the maximum conversion and yield are achieved at the lowest temperatures and highest pressures, whereas minimum values occur at higher temperatures and lower pressures. The reactant ratio also has a significant influence on these two parameters, particularly up to a ratio of 4; beyond this, the difference between ratios 4 and 5 is often negligible, suggesting that using a higher ratio might not be economically justified given the high cost of hydrogen.

**Figure 3:**
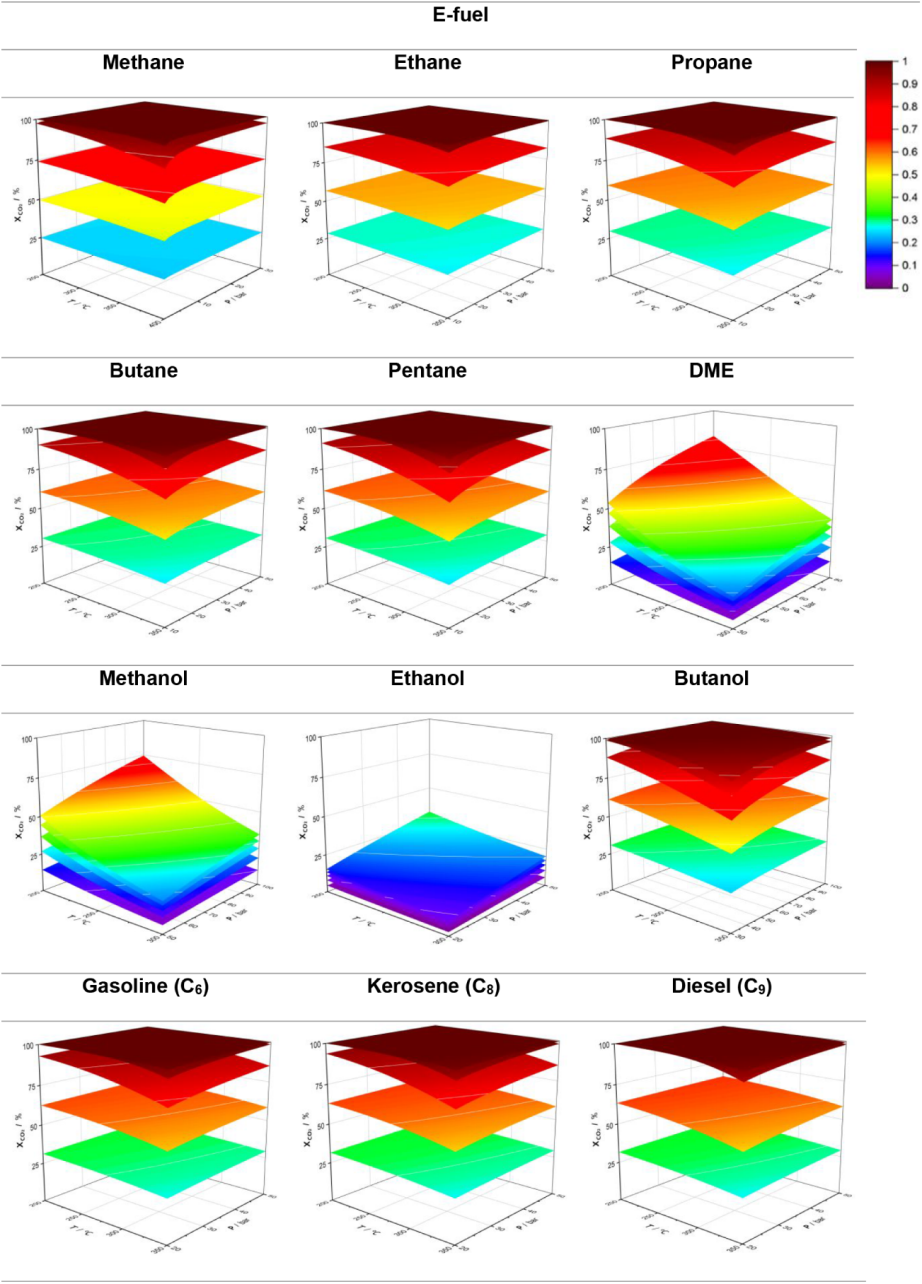
Influence of temperature and pressure on the conversion of different e-fuels.

**Figure 4:**
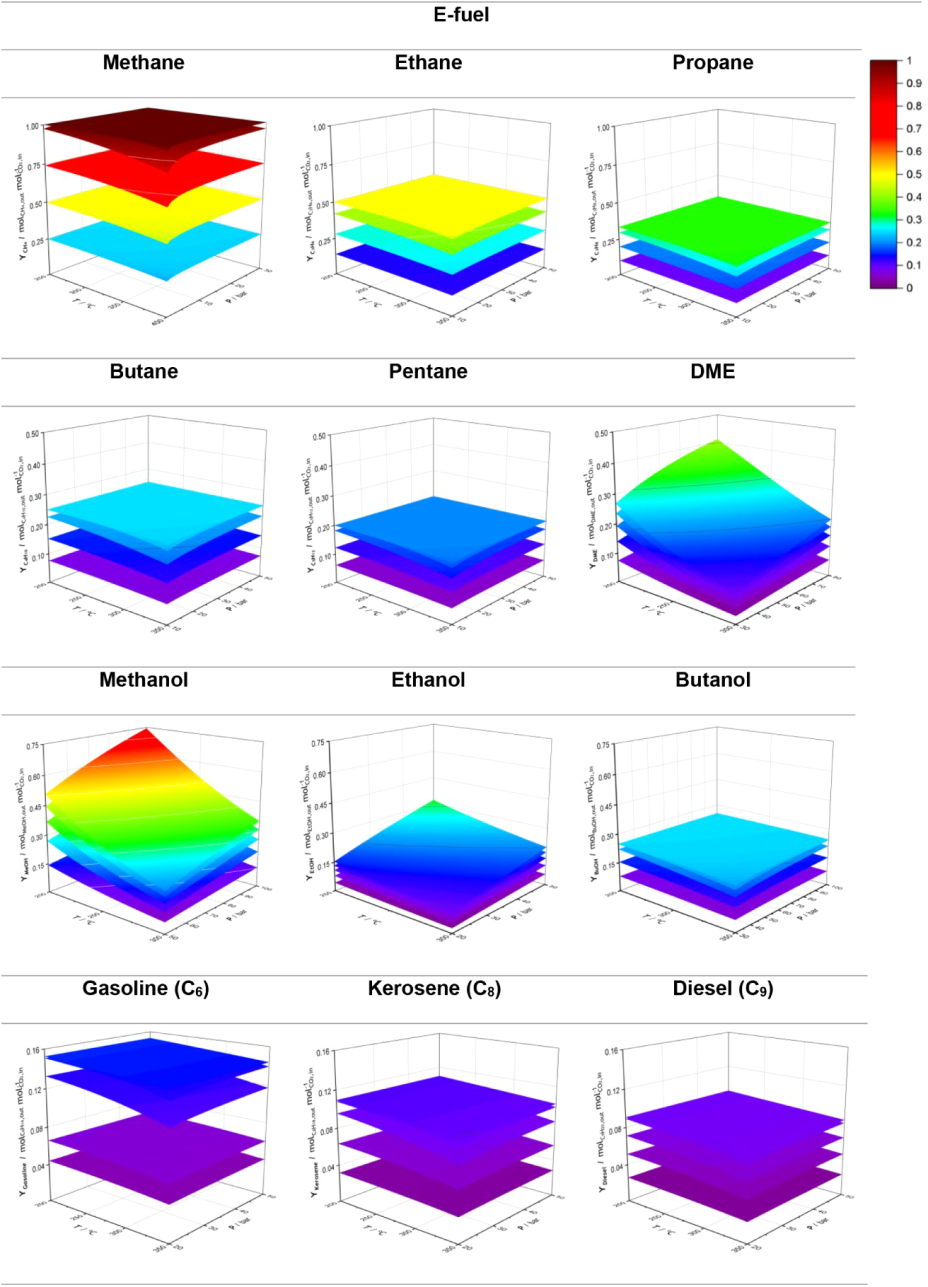
Influence of temperature and pressure on the yield of different e-fuels.

For a H_2_/CO_2_ ratio of 4, methanation is complete under most tested conditions, except for the cases involving lower pressure and higher temperature (cf. Figure 4). Under such conditions, a small fraction of CO can be formed via an endothermic RWGS reaction. Complete methanation at this ratio is further supported by the observation that the outlet molar flow of water is twice that of methane, consistent with the methanation reaction. Higher hydrocarbons, from ethane to pentane, are normally produced at a H_2_/CO_2_ ratio of 3. When simulated individually, these compounds present a progressive decrease in conversion and yield as the carbon chain length increases. Thermodynamically, methane is by far the most stable hydrocarbon; as the chain length increases, stability decreases, making equilibrium and Gibbs free energy minimization more difficult. Overall, the CO_2_ conversion and yield stabilize at maximum values that differ for each fuel. A similar trend is observed for representative molecules of gasoline, kerosene, and diesel-range hydrocarbons, specifically and respectively, C_6_H_14_, C_8_H_18_, and C_9_H_20_.

Methanol and ethanol show similar tendencies, but the shape produced is different than that of hydrocarbons: the slopes increase with the decrease in T and increase in P, contrary to what happens with hydrocarbons, which means these changes significantly impact the conversion and yield of these alcohols. Butanol, however, displays a trend more aligned with that of hydrocarbons. It seems that methanol and ethanol are less sensitive to pressure than butanol, which can be attributed to the differences in mole variation between reactants and products. As shown in Eqs 24 and 25, CO_2_ hydrogenation to methanol involves a stoichiometric mole change of −2, while butanol formation involves −8. This justifies butanol formation being more responsive to pressure, thereby explaining the lower yields observed under low-pressure conditions.

24 $$CO_{2}+3H_{2}⇌CH_{3}OH+H_{2}O,\Delta H_{r,298K}=-49kJ\cdot mol^{-1}$$

25 $$4CO_{2}+9H_{2}⇌C_{4}H_{9}OH+4H_{2}O,\Delta H_{r,298K}=-416kJ\cdot mol^{-1}$$

The work by Miguel et al.^57^ studied the methanation and methanol formation via CO_2_ hydrogenation, further supporting the distinct trends observed between these two routes. For a certain, unspecified H_2_/CO_2_ ratio, the study confirmed that the decrease in temperature and increase in pressure led to higher carbon dioxide conversions, which is consistent with the simulation results obtained. Specifically, methane production reaches a plateau, with conversion values stabilizing at a maximum, while methanol conversion continues to rise under the same conditions. Their work has also been able to successfully conduct CO_2_ methanation with nearly 100% selectivity. Figure 5 demonstrates the maximum (HY) and minimum (LY) yield values for each e-fuel, along with the corresponding operating conditions (temperature, pressure, and H_2_/CO_2_ ratio). This figure provides a more intuitive understanding of the different operating zones for each e-fuel, which were subsequently used in the integrated approach (Section 3.2). As shown in Figure 5, the yield decreases progressively with increasing chain length, from methane to pentane, and from gasoline- to diesel-range HCs. In contrast, no general trend can be derived for alcohols, as their yield depends strongly on component-specific conditions. Even though it is expected that the lower yield cases turn out to be economically unfeasible, it is of research interest to evaluate the thermodynamic and economic trade-offs between the two scenarios.

**Figure 5:**
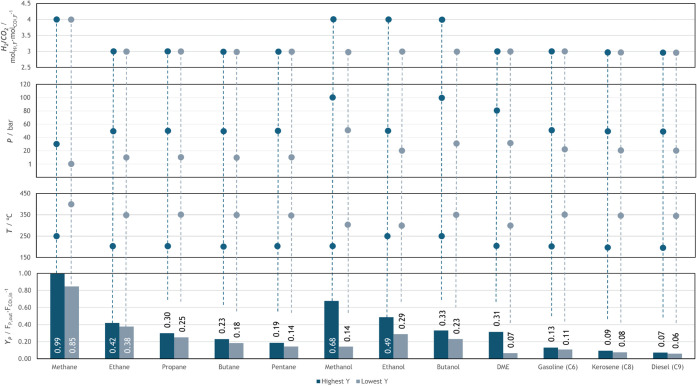
Conditions for the highest and lowest yields of each e-fuel in terms of H_2_/CO_2_ ratio, temperature, and pressure.

### 3.2 Integrated Approach—Techno-economic Analysis

In the integrated approach of this work, the reactor block was merged into a complete and more universal flowsheet applicable to each e-fuel. Before specifying the costs of individual process units, the analysis considers only the revenue from e-fuel sales, operational expenses (OPEX), and hydrogen cost. Figure 6 presents the production-phase rentability of the different e-fuels, based on the flowsheet depicted in Figure 2 and considering both highest and lowest yield scenarios. The results indicate that only gasoline (in both conditions) and diesel (under the highest yield conditions) exhibit economic potential for e-fuel production via CO_2_ hydrogenation. The corresponding positive α values for these potentially viable processes are summarized in Table 6.

**Figure 6:**
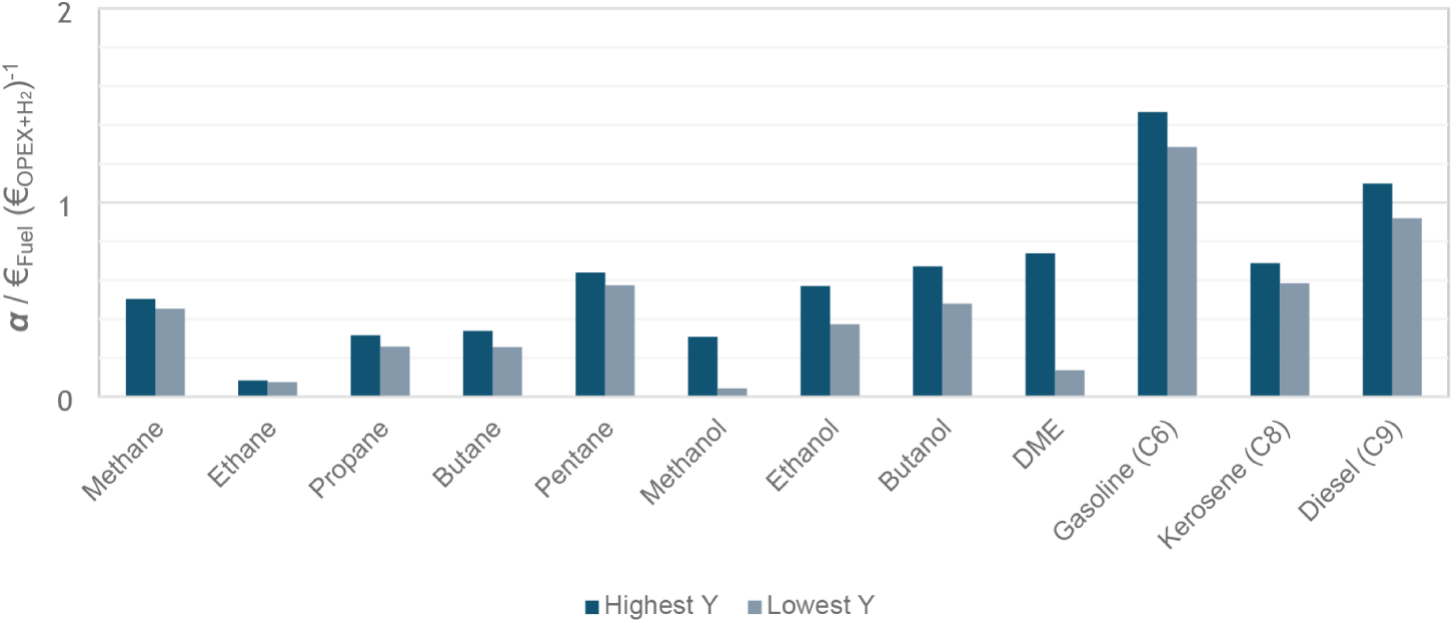
Production phase rentability (α) of the different e-fuels.

**Table 6:** Summary of Positive Production Phase Rentabilities in This Study

	*T* = 200 °C, *P* = 50 bar		*T* = 350 °C, *P* = 20 bar
e-Fuel	Gasoline (C_6_)	Diesel (C_9_)	Gasoline (C_6_)
α/€_Fuel_·(€_OPEX+H2_)^−1^	1.47	1.10	1.29

Although the economic viability of e-fuel production has long been debated, costs are likely to exceed the revenue due to the absence of heat integration, a strategy not addressed in this work. Heat integration improves energy efficiency by reducing the dependence on external utilities, which could significantly lower the consumption of electricity, cooling and chilled water, and refrigeration, thereby enhancing the rentability of the process. Additionally, low rentabilities may also stem from pricing inaccuracies or inadequacies. Price inaccuracy arises from significant regional and temporal fluctuations, while inadequacy stems from using market prices associated with conventional fossil fuels, that is, produced from environmentally damaging processes, not accurately reflecting the true market value of their synthetic analogues. For instance, methane [α = (0.50, 0.35)], despite exhibiting the highest yield values, remains far from having an economically viable production. This can be attributed to its low market price, driven by its abundance and its widespread use for heating. In addition, the acquisition cost is very close to the sale price, leaving minimal margin for rentability. Finally, pressurization of the methane for grid injection would also likely be a significant additional cost. Ethane [α = (0.08, 0.08)], while thermodynamically promising, is often found as a component of natural gas and is rarely used as a standalone fuel; thus, the pricing potentially does not reflect its true value in an e-fuel context. Propane [α = (0.32, 0.26)] and butane [α = (0.34, 0.26)] are more commonly used for various household applications and sold directly to consumers at prices between 2 and 3 €·kg^–1^. For comparison, in Portugal, a 45 kg cylinder of Rubis Gás propane sells for 109.80 € (2.44 €·kg^–1^), while 13 kg of butane from the same brand retails for 34.60 € (2.66 €·kg^–1^). At these retail price points, the corresponding rentability parameters would exceed 1 (Table 7), indicating a potentially stronger economic case and suggesting room for improvement and optimism regarding the viability of these e-fuels. However, the scope of this study aims to compare the resulting hydrogenation production costs to conventional ones.

**Table 7:** Alternate Rentability Parameters for Household Gas Production

	*T* = 200 °C, *P* = 50 bar		*T* = 350 °C, *P* = 10 bar	
e-Fuel	Propane	Butane	Propane	Butane
α/€_Fuel_·(€_OPEX+H2_)^−1^	1.41	1.56	1.15	1.17

Pentane [α = (0.64, 0.57)], despite not showing potential for viability improvement, can contribute positively when blended with gasoline, which, under the conditions evaluated in this study, shows more promise as an e-fuel.

Gasoline [α = (1.47, 1.29)] consistently presents satisfactory values of α for both the best and worst yield levels, indicating its potential viability across all conventional production scenarios.

Diesel [α = (1.10, 0.92)] appears viable only in the highest yield scenario, despite having OPEX and CAPEX figures similar to those of gasoline, since diesel is cheaper than gasoline.

Kerosene, [α = (0.69, 0.58)], despite ongoing interesting e-kerosene projects across Europe, has proven economically unviable under the assumed pricing conditions. This outcome further enforces the limitations of this analysis and the importance of context-specific and policy considerations when assessing e-fuel feasibility.

Finally, methanol [α = (0.31, 0.04)], ethanol [α = (0.57, 0.37)], butanol [α = (0.67, 0.48)], and DME [α = (0.74, 0.14)] exhibit significant viability challenges due to limited yield levels and comparatively low market prices, both of which restrict their economic feasibility under the assessed conditions.^58^

For gasoline (both temperature and pressure conditions) and diesel (highest pressure and lowest temperature conditions), the CAPEX was estimated. CAPEX includes the acquisition costs of the compressors, heat exchangers, the reactor, and the condenser, as detailed in Table 8. Compressors make up 61–67% of total CAPEX, while the reactor makes up approximately 30%. The remainder is associated with the heat exchangers.

**Table 8:** Capital Expenditure for the Positive Production Phase Rentability Cases

	CAPEX/k€		
	Gasoline (C_6_)		Diesel (C_8_)
Operating conditions	*T* = 200 °C, *P* = 50 bar	*T* = 350 °C, *P* = 20 bar	*T* = 200 °C, *P* = 50 bar
Compressors	858	944	858
Heat exchangers	86	134	86
Reactor	417	417	417
Condenser	54	50	54
*Total*/M€	1.41	1.54	1.41

The revenue considered in this analysis derives exclusively from the production of e-fuels, since no other resulting streams were treated in nonscaled equipment for further reprocessing. As seen in Figure 7, the cumulative cash flow is constantly decreasing. Given that it remains negative throughout the analysis period, the slope indicates a nonexistent IRR and payback time. Consequently, the NPV values for each process confirm the economic unviability of the studied cases under current assumptions. The NPV values are listed in Table 9.

**Figure 7:**
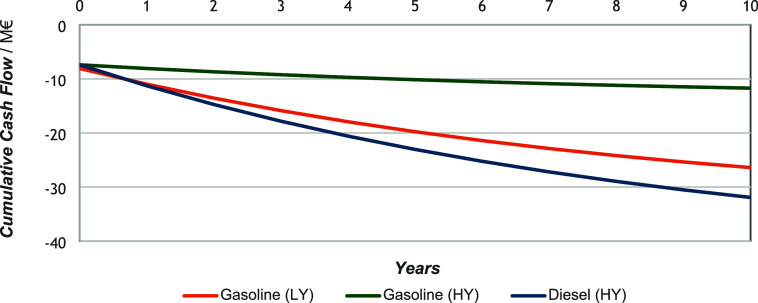
Cumulative cash flow throughout 10 years of production.

**Table 9:** Net Present Values after 10 Years

NPV/M€		
Gasoline (HY)	Gasoline (LY)	Diesel (HY)
–0.23	–0.94	–1.38

The NPV remained negative for all cases. Among the major cost contributors, hydrogen purchase is one of the most significant expenses, exerting a substantial influence on overall profit alongside the selling price of the e-fuel. A reduction in the hydrogen price or an increase in fuel price could, therefore, enhance economic revenue. To evaluate these effects, a sensitivity analysis focusing on the hydrogen cost and a search for the minimum viable selling price of the e-fuel cost were conducted.

Figures 8, 9, and 10 show the influence of varying hydrogen costs on profitability, covering a range from 1 €·kg^–1^ to 3 €·kg^–1^ (the base case), with increments of 0.5 €·kg^–1^. Obviously, diesel remains the least economically favorable, followed by gasoline (under LY and HY conditions, in that order). According to Figure 8, if hydrogen were priced at 1.5 €·kg^–1^, a return on investment could be achieved within 5 years and in just 3 years if the price dropped to 1 €·kg^–1^. Even at 2 €·kg^–1^, the positive slope suggests that economic viability could potentially be reached over a longer time frame, i.e., 10+ years.

**Figure 8:**
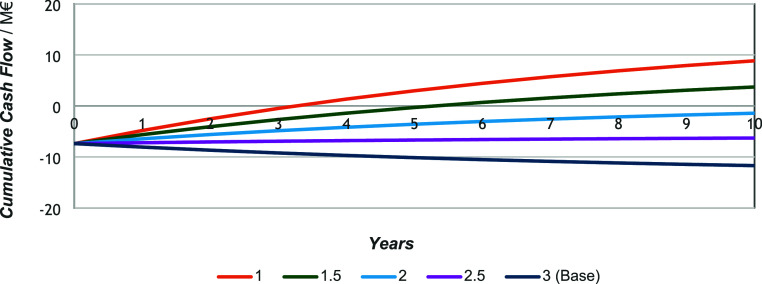
Gasoline (HY) cash flow variation with variation in hydrogen cost.

**Figure 9:**
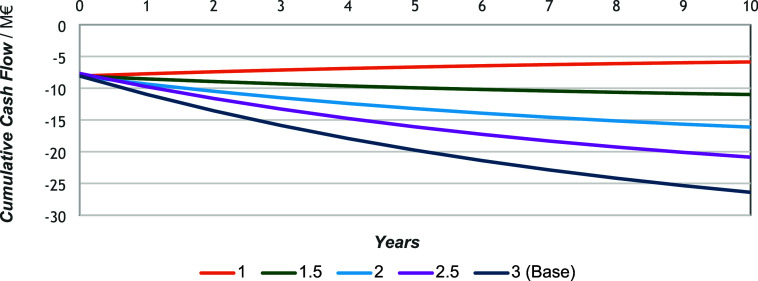
Gasoline (LY) cash flow variation with variation in hydrogen cost.

**Figure 10:**
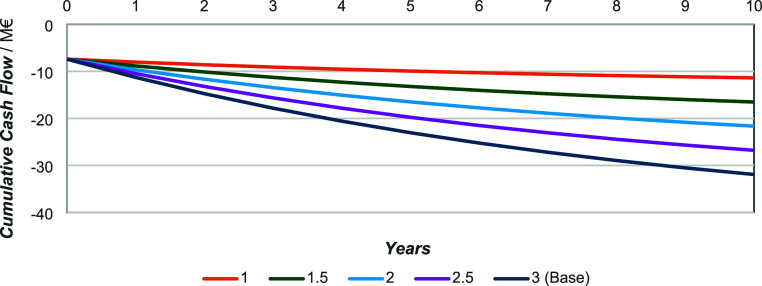
Diesel (HY) cash flow variation with variation in hydrogen cost.

Gasoline (Figure 9), under the least favorable yield conditions and with hydrogen going for 1 €·kg^–1^, could be profitable. However, the payback period would exceed 10 years and remains highly uncertain, making the investment unattractive from a conventional economic standpoint. In contrast, as shown in Figure 10, diesel demonstrates no indication of being viable under any of the considered hydrogen pricing scenarios.

While the majority of the results indicate a decrease in cumulative cash flow, the scenarios showing a positive one might hold promise. As stated in Section 1.1, projections suggest that by 2050, green hydrogen could cost 1–1.5 €·kg^–1^ and 2 €·kg^–1^ in designated regions.^44^

For the normal cost of hydrogen, the minimum e-fuel selling price, *C*_min_, was determined with the help of “Solver”, the iterative calculation method in Microsoft Excel. Table 10 shows the reached minimum costs, conventional costs, and how much the percentual variation between both is.

**Table 10:** Minimum and Conventional Price Points for e-Fuels Depending on Production

	Gasoline (HY)	Gasoline (LY)	Diesel (HY)
*C*_min_/€·kg^–1^	3.17	3.37	4.00
*Price*/€·kg^–1^	2.09	2.09	1.65
*Variation*/%	52	61	142

From the variations in price, it is visible that these prices are not competitive with those of conventional fossil fuels. Besides that, the real variation is probably larger since the purification of the e-fuels and storage must be considered in the overall expenditure. For comparison, other studies on specific e-fuels have shown similar conclusions when it comes to the future reduction in hydrogen and, therefore, fuel price.^58,59^

Although these results are valuable, further investigation and considerations are needed. Realistically, there are more variables and operation units in action, and each process should be modeled in a specific manner when it comes to separation unit, requirements, care, and catalyst, another component whose costs were not considered. Therefore, a real e-fuel plant might end up costing a lot more than that calculated in this study. In the future, it is also recommended to consider oxygen and water vapor in the flue gas composition, as extra caution and attention to specifics may be necessary. For instance, since the reaction between hydrogen and oxygen is explosive, a separation process for flue gas might be needed in the form of pressure swing adsorption (PSA), for instance.

In addition, for compatibility with existing motors and systems, some e-fuels like e-gasoline and e-kerosene should respect the composition requirements. Though utilities are not the majority of costs, it is always helpful to study the effect of heat integration, not from a thermodynamic or energy efficiency perspective, but from a resource-saving one. Finally, after secondary separation, it should also be worth it to have a recycling of any remaining reactant. Even though conversion of CO_2_ was of great order, there should be an effort to recycle it and make the use of e-fuels as aligned with the carbon-neutral goal as possible. H_2_ shows a greater outlet flow, and since it is expensive, it is also very worthy of being repurposed. Future work should be focused on the development of detailed, industrially representative e-gasoline production flowsheets, including rigorous process simulation, heat integration, and quantification of H_2_ losses due to conversion limitations over multiple passes. Additionally, the economic and environmental trade-offs between not recycling hydrogen and the additional compression and cooling duties associated with H_2_ recycling should be systematically assessed.

Finally, it must be noted that the real cost of fossil exploration lies in environmental, social, and carbon costs. Energy independence, mainly nowadays, on the brink of possible wars between oil behemoths, is more important than ever. Investing in a country’s hydrogen economy and using and repurposing it inside the country leads to a crucial circular economy and energy autonomy. Even if gray hydrogen, from steam methane reforming, is used, it is still a positive step toward decarbonizing society and offsetting the oil industry’s mistakes. It is more likely than not that the future holds taxation and fining laws on fossil fuels; so, instead of being taxed and fined later, governments and oil companies need to invest as soon as they can.

## 4 Conclusion

The present work focused on studying the thermodynamic and economic potential of the hydrogenation of CO_2_ from polluted/exhaust streams to obtain cleaner fuels in the form of electrofuels. The aim of producing and utilizing e-fuels is to benefit the environment and efficiently use energy, positively impacting society. For this, a thermodynamic analysis on an equilibrium reactor was simulated, and potential viability from OPEX, hydrogen cost, and conventional fuel price alone was evaluated. Then followed the study of a more complex and integrated process, ending in a techno-economic analysis of e-fuel production plants for the three promising cases. This techno-economic analysis was carried out for potentially profitable situations.

The thermodynamic equilibrium study helped figure out carbon dioxide conversion and fuel fractional yield in the conventional production ranges of temperature, pressure, and feed H_2_/CO_2_ ratio. With a decrease in temperature and an increase in pressure and H_2_/CO_2_ ratio, both parameters showed growth, whose degree depends on the reactant and product mole difference, thermodynamic stability, and enthalpy change. These variables also showed an effect on the shape of the conversion vs *T* and *P* surfaces. At best, methane and methanol showed the highest yield values, at 1 mol_CH4,out_·mol_CO2,in_^–1^ and 0.75 mol_MeOH,out_·mol_CO2,in_^–1^, showing high thermodynamic stability.

After, the integrated approach study was carried out. Essentially, costs of feedstock, energy, and utilities were considered for an initial viability assessment, with the assumption that e-fuels could be sold at the price of conventional fuel. This proved, for the most part, to be economically detrimental. The exceptions were e-gasoline/e-hexane (HY, α = 1.47, and LY, α = 1.29) and e-diesel/e-nonane (HY, α = 1.10), where conventional fuel price sales surpassed operational and feedstock costs. For these cases, capital expenditure was considered.

In the final phase, CAPEX, namely the cost of operation units, and other parameters such as workforce and maintenance were applied to predict cash flow evolution. For the base case, considering hydrogen purchase at 3 €·kg^–1^, no process showed viability. For the current hydrogen cost, e-gasoline would have to be at least 1.5 times more expensive than conventional gasoline, while e-diesel should cost at least 2.4 times more than fossil diesel. After analyzing the sensitivity of cash flow to the reduction in hydrogen price, gasoline showed possible viability for the range of 1–2 € per kg of H_2_, in 3 to 10+ years, while diesel only for 1 € per kg of H_2_, but with an unpredictably large payback time. While nowadays, these are prices of the environmentally damaging gray hydrogen, prospects in studies preview the reduction of green hydrogen price to that range until 2050, making this technology very promising.

## Glossary

CAPEX: Capital Expenditure
CAS: Chemical Abstracts Service
CEPCI: Chemical Engineering Plant Cost Index
DAC: Direct Air Capture
DME: Dimethyl Ether
FTS: Fischer–Tropsch Synthesis
HC: Hydrocarbon Compounds
HY: Highest Yield
IAPMEI: Agency for Competitiveness and Innovation
IRR: Internal Rate of Return
LY: Lowest Yield
NPV: Net Present Value
OPEX: Operational Expenditures
PSA: Pressure Swing Adsorption
RWGS: Reverse Water–Gas Shift
